# Thermally Activated Sliding of C_60_ on Gold

**DOI:** 10.1021/acsomega.5c02993

**Published:** 2025-06-10

**Authors:** Matteo Pierno, Lorenzo Bruschi, Guido Paolicelli, Alessandro di Bona, Stefania Benedetti, Nicola Manini, Andrea Vanossi, Giampaolo Mistura

**Affiliations:** † Dipartimento di Fisica e Astronomia “G. Galilei”, Università di Padova, via Marzolo 8, 35131 Padova, Italy; ‡ CNR, Consiglio Nazionale delle Ricerche-Istituto Nanoscienze-Centro S3, via Campi 213/a, 41125 Modena, Italy; § Dipartimento di Fisica, Università di Milano, via Celoria 16, 20133 Milano, Italy; ∥ CNR-IOM, Consiglio Nazionale delle Ricerche - Istituto Officina dei Materiali, c/o SISSA, Via Bonomea 265, 34136 Trieste, Italy; ⊥ International School for Advanced Studies (SISSA), Via Bonomea 265, 34136 Trieste, Italy

## Abstract

Gold nanoclusters are known to slide easily on a graphite surface. In this study, we confirm the slipperiness of the gold–carbon interface by studying the sliding behavior of fullerene adsorbates on gold by using a quartz crystal microbalance (QCM). More precisely, we transfer high-quality gold electrodes deposited on an atomically flat mica substrate to the QCM. By means of an effusion cell, we deposit C_60_ molecules on the QCM gold electrode kept in ultrahigh vacuum. We observe the pinning of the fullerene adsorbates at room temperature. As the temperature increases above 320 K, the fullerene adsorbates begin to slide. This thermally activated sliding is explained in terms of a simple diffusive model.

## Graphical abstract

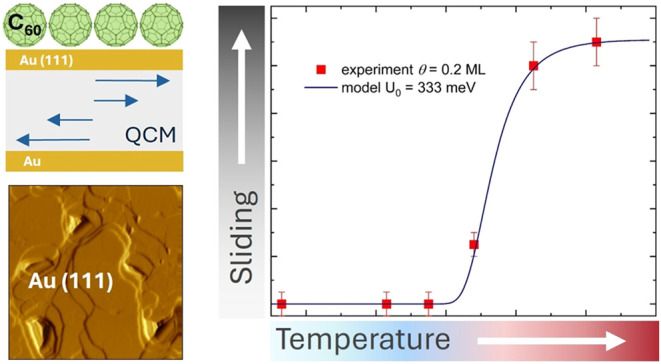

## 1 Introduction

Although the empirical laws of macroscopic friction are well-known,^1,2^ the fundamental understanding of the tribological phenomena at the smallest scales is still lacking from many points of view, due to the difficulty in probing systems with many degrees of freedom under an often ill-characterized, buried interface, where strong nonlinear dissipative dynamics takes place, coupled with a basic lack of fundamental physical theory.^3^

In this context, a number of fundamental challenges remain to be addressed and several opportunities exist. In recent years, extensive research has been conducted on the potential for manipulation of nano- and mesoscale friction and mechanical responses across various physical systems. For example, the possibility of varying friction, e.g., by means of temperature-controlled transitions such as the charge density wave,^4^ the metal–insulator^5^ and metal-superconductor^6–8^ transitions have been investigated in some detail, with interesting outcomes. Field-driven structural changes^9^ or charged species^10–12^ also exploit similar ideas. Notably, in nanoscale proximal probe experiments^13–15^ and in simple atomic physisorbed systems, such as rare gases on metallic surfaces,^16,17^ the tribological properties are directly influenced by temperature itself, possibly demonstrating a consistent ″thermolubric″ behavior, where friction decreases as temperature rises. The underlying reason for this typical pattern is that random thermal fluctuations help the sliding interface overcome interlocking barriers, thus facilitating movement. Whether such temperature-induced lubricity still persists for “heavier” molecular adsorbates certainly needs further investigation.

It is well-known that the gold–carbon interface is characterized by low friction. This has been verified in a variety of systems, including gold nanoclusters,^18–20^ and gold islands^21,22^ on graphite, graphene nanoribbons on gold surfaces,^23–25^ fullerene-wheeled single molecular nanomachine on gold.^26^ In particular, fullerene C_60_ is a “soccer ball”-shaped molecule whose spherical structure, chemical stability, and rotational motion in the bulk suggest that it could be highly effective as a lubricant.^27,28^ The idea that rolling C_60_ molecules could act as “nanobearings” has been repeatedly raised, mostly without experimental confirmation.^29^ Nanotribology studies of Krypton and Xenon monolayers carried out at cryogenic temperatures with the quartz crystal microbalance (QCM) also considered metallic electrodes coated with C_60_ molecules.^30,31^ In particular, to explore whether there was a lubricating effect, sliding friction of methanol monolayers on fixed and rotating C_60_ layers was measured with a QCM at room temperature.^32^ Surprisingly, increased friction was observed for the case of rapid rotation, contradicting the expected “nanobearing” effect.^32^ Similarly, the orientational order–disorder phase transition of a fullerite crystal, in which the free rotation of C_60_ at high temperature is reduced to a low temperature hindered rotation, was found to cause an abrupt change in friction measured with an atomic force microscope.^33^ However, this change in friction force was quantitatively consistent with the observed change in adhesion.^33^ Therefore, the C_60_ rotation did not provide an additional energy dissipation channel in the friction process.

More recently, the superlubricity of fullerene derivatives was achieved by the construction of regular host–guest assembly structures on graphite.^34^ Comprehensive studies of C_60_ adsorption on the Au(111) surface^35^ have also indicated that C_60_ molecules promote the diffusion of highly mobile vacancies in the upper layers of the gold substrate near room temperature, possibly affecting the friction of the C_60_ adsorbate. As a preliminary step to unravel the possible temperature-induced lubricity of molecular adsorbates, we have carried out a dedicated study exploiting the quartz microbalance technique where C_60_ molecules were deposited on the QCM gold electrode in an extended temperature range, from approximately 273 to 373 K. The present work indeed reports a dramatic temperature dependence of the friction of C_60_ adsorbates on gold: we observe no slippage at room temperature or below, while at higher temperature a thermolubric sliding of the C_60_ adsorbate is found instead and interpreted via a simple modeling. The paper is organized as follows: after the Introduction, the preparation of the gold surfaces and the description of the QCM are presented in Materials and Methods, together with the theoretical modeling. The measured slip times of C_60_ taken at different temperatures are then discussed and compared with the theoretical modeling in Results and Discussion.

## 2 Materials and Methods

The two faces of the quartz crystal were optically polished and covered with gold keyhole electrodes by the supplier. For this study, we specially prepared one of the electrodes to obtain an atomically flat Au(111) surface under air conditions. A specific protocol consisting of two distinct steps was optimized to achieve this goal. First, a gold layer (70–100 nm) was thermally evaporated on an atomically flat mica disk on a dedicated deposition chamber. High-purity gold fragments (Kurt J. Lesker Company, Au 99.99% pure) were used as evaporation material. The mica substrate was kept at 623 K during deposition, which took approximately 20 min. The sample was cooled in the deposition chamber and finally extracted into air to proceed with the final deposition phase. In the second step, the gold layer supported by the mica substrate was placed in contact with the commercial QCM gold keyhole electrode inside a homemade, temperature-controlled pressure cell. We transferred the gold film from the mica to the QCM electrode by applying a combination of high pressure and temperature.^36,37^ The pressure cell was initially heated to 473 K, then a pressure of up to 28 MPa was imposed by a pneumatic actuator, and the system was held for about 60 min under these conditions. The cell was naturally cooled to room temperature, and then the system was slowly unloaded. At the end of this process, the evaporated gold film on the mica was completely transferred onto the QCM electrode, so that its new external surface, resulting from the detachment of the mica itself, was extremely clean and crystalline. Temperature, pressure, stamping time, as well as details on the first gold mica deposition, were carefully optimized, and the typical results of this procedure are illustrated in Figures 1 and 2.

**Figure 1:**
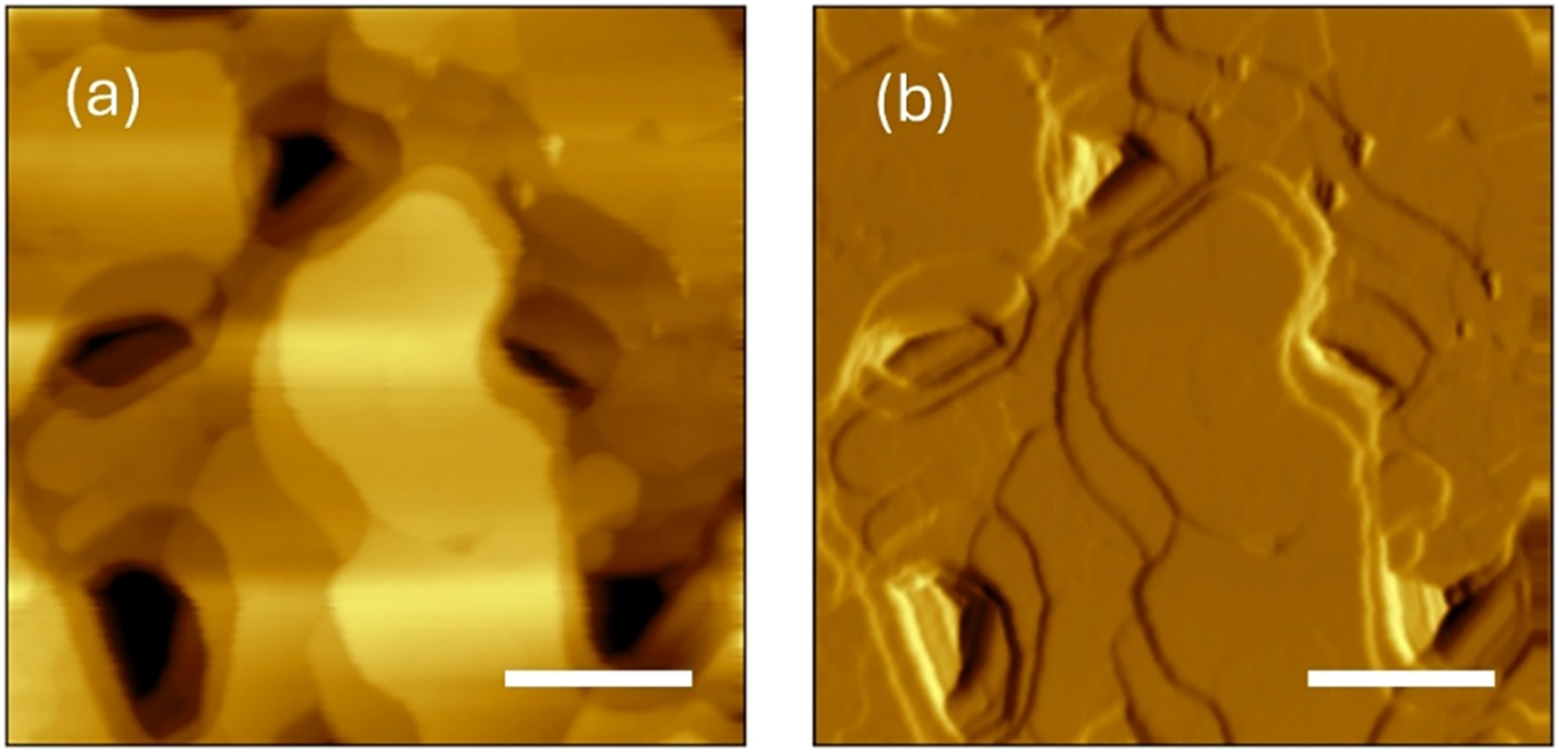
(a) AFM image of the surface of gold directly transferred on the gold electrode (1 × 1 μm^2^); (b) Derivative of the height signal of the panel (a). The scale bars are 250 nm.

**Figure 2:**
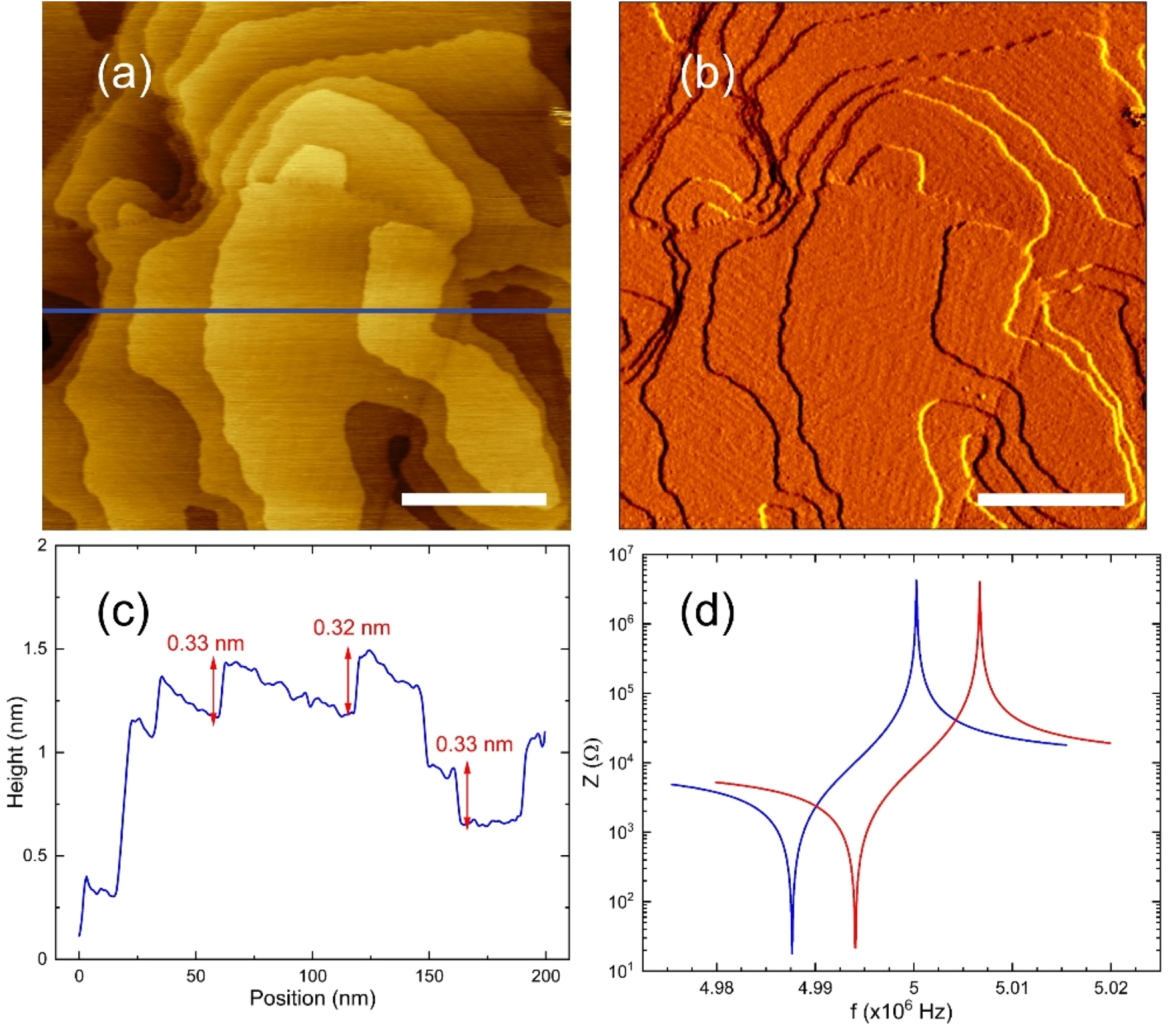
(a) STM image of the gold surface transferred to the QCM electrode. The terrace steps are monatomic and the total height range is about 2 nm. (b) The derivative signal, although characterized by a low contrast, highlights the “herringbone” reconstruction of the Au(111) surface. The scale bars 50 nm. (c) Height profile along the blue line in panel (a), showing the atomic step. (d) Frequency spectrum of the electrical impedance *Z* of the quartz before (red) and after (blue) the transfer procedure. The corresponding *Q* values are 100780 and 105500 respectively.

Figures 2a and b show an STM image obtained under UHV conditions of the modified gold electrode. The lateral size (200 nm) of this image is similar to that of the terraces identified in the AFM image. The total height range is about 2 nm, and the monatomic steps are clearly visible in Figure 2c. The gold film was characterized by XPS spectroscopy. The spectra, measured directly on the gold surface without applying any UHV cleaning procedure, present the complete XPS signature of a clean gold surface with tiny traces of oxygen and a very small presence of carbon. Although we cannot rule out that they may somewhat affect the precise values of the measured slip times, we expect that the observation of thermally activated sliding of fullerene monolayers on the gold surface will not be qualitatively altered. The electrical impedance *Z* of the QCM plotted in Figure 2d, monitored before and after the transfer, exhibits an appreciable downward frequency shift, associated with the transferred gold mass. However, the resonance quality factors *Q* remain unchanged, demonstrating a very good adhesion between the gold components of the electrode and the quartz substrate.

### 2.1 Quartz Crystal Microbalance Setup

For the measurement of the friction of C_60_ films, we used AT cut quartz disks of diameter 8.9 mm and thickness 0.32 mm. Their two opposite faces were driven in fundamental mode with resonance frequency *f*_0_ ∼ 5 MHz using a frequency modulation technique.^17^ At resonance frequency, the two opposite faces of the quartz disc oscillated in a transverse shear motion, as shown in Figure 3a. The quartz crystals exhibited quite sharp resonances, characterized by *Q* quality factors comprising between 20,000 and 100,000. As an example, the normalized resonance curve in Figure 3b refers to a quartz crystal driven in vacuum at a temperature *T* = 330 K. The continuous red line is the nonlinear fit to the data according to the formulas previously reported:^28^ the resulting quality factor is *Q* ∼ 70,000.

**Figure 3:**
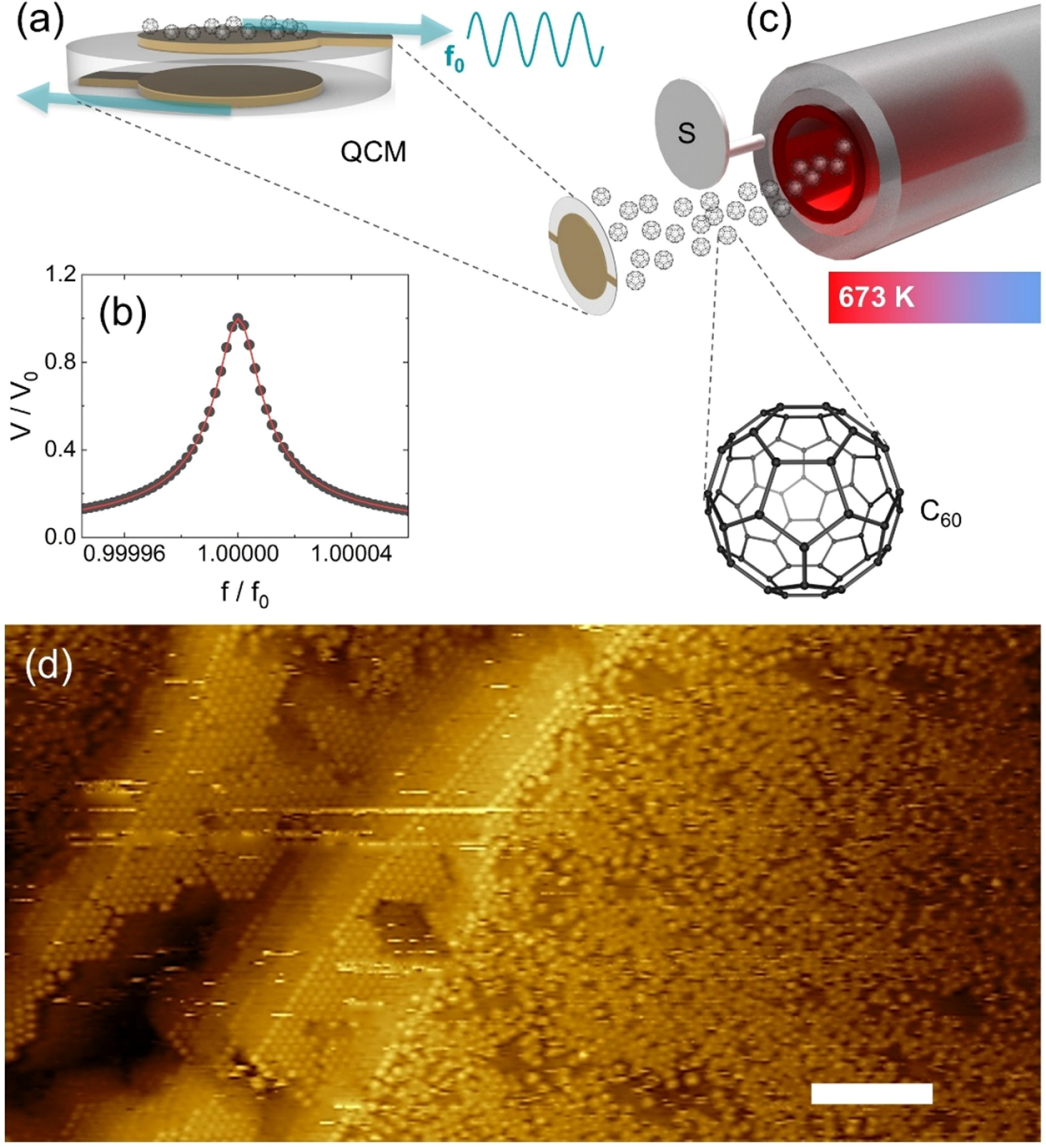
(a) Sketch of the QCM oscillating in the fundamental shear mode. (b) Normalized resonance curve of a quartz crystal. (c) Schematic of the evaporation process: high-purity C_60_ powder is loaded into the crucible and sublimed at *T* = 670 K. Opening the shutter S starts the dosing of C_60_ on the QCM. (d) STM image showing clustering of C_60_ in proximity of crystal edges. The scale bar is 10 nm.

The quartz crystals were mounted in a custom case and inserted into an ultrahigh-vacuum chamber specially made for the QCM experiments.^38^ The copper sample cell was thermally anchored to a Peltier cell housed in a receptacle outside the chamber.^38^ Thus, it was possible to vary the QCM temperature between 263 and 373 K, with a temperature stability better than 0.1 K for 1 day, which was much longer than the typical data acquisition time of approximately 1 h.

The 99.9+% pure C_60_ from Sigma-Aldrich (Merck Group, Germany) was sublimed from an organic material effusion cell (series OEZ made by Dr. Eberl MBE-Komponenten GmbH, Germany) housed in the UHV chamber at a distance of about 30 cm from the quartz electrode, see Figure 3c. The design of cell temperature hotspots is such that overshooting is effectively eliminated; therefore, decomposition of the organic material is prevented. The temperature of the effusion cell was controlled with stability of ± 0.05 K in a range of 320 to 1100 K, allowing precise control of the deposition rate.

For the deposition of C_60_ molecules, the temperature of the effusion cell was slowly increased to 770 K with the shutter always closed. The gun temperature was then stabilized at 623 K for about 20 min before opening the shutter. Once the deposition was completed, the quartz crystal had to be replaced because it was not possible to clean the surface from the C_60_ molecules scans. In fact, the typical temperature required for the desorption of the first C_60_ monolayer^26^ (well above 800 K) is close to the Curie temperature of the quartz, *T*_C_ = 846 K.

### 2.2 Modeling

The QCM oscillation applies an inertial force to each C_60_ molecule, which, given the amplitude and oscillation frequency, we estimate in the 10^–19^ N region at its peak. According to ab initio estimates^39,40^ of the lateral energy barrier encountered by an isolated C_60_ when translating in contact with a flat Au(111) surface, a plausible barrier value in the *U*_1_ ∼ 100 meV region indicates that the barrier-crossing force ∼*πU*_1_/*a* ∼10^–10^ N far exceeds the QCM driving, indicating that the latter acts only as a weak perturbation of the thermal diffusion of C_60_ on the surface.^41^ This very weak forcing regime has been addressed by linear-response methods in ref 42. However, given, on the one hand, the complications of the Au(111) surface, involving the concrete possibility that surface layer Au vacancy diffusion affects the frictional properties of the C_60_ layer above,^35^ and, on the other hand, the nontrivial roles of molecular rotations and deformations, it would be extremely problematic to apply that level of theory to predict the slip time of the C_60_/Au­(111) interface.

For this reason, we resort to a very basic model. First of all, at finite coverage, the C_60_ molecules aggregate in medium-sized islands, whose pinning barrier *U*_0_ against translation is expected to exceed the single-molecule barrier *U*_1_. Let us indicate with τ_diff_^–1^ the average rate at which one C_60_ molecules crosses a lateral barrier, and with $x=\frac{\tau_{\mathrm{diff}}^{-1}}{f}$ the average number of barrier crossings per QCM oscillation period. When *x* ≪ 1 thermal jumping is suppressed, to the point that the dissipation, and the resulting slip time, measured in our experiment, are negligible. When *x* ≫1 diffusion occurs frequently, leading to a significant broadening of the QCM resonance and, correspondingly, to a growth of the measured slip time τ_s_. We can model the resulting slip time as a constant τ_c_ multiplied by a sigmoid-type function that increases rapidly from 0 to 1 as *x* increases from 0 to exceed unity, for example $s\left(x\right)=\frac{2}{1+\exp\left(x^{-4}\right)}$ . We point out that the precise functional dependence of *s*(*x*) is really unimportant, as long as it starts very flat near the origin and it grows rapidly toward unity. We tested several s-shaped functions and the results are practically unchanged; see next section.

We postulate a thermally activated barrier crossing rate $\tau_{\mathrm{diff}}^{-1}=\tau_{0}^{-1}e^{-U_{0}/K_{B}T}$ , where τ_0_^–1^ indicates the attempt rate. Inserting this expression into the definition of *x*, we obtain a simple model for the rise of the slip time τ_s_ = τ_c_ *s*(*x*) as temperature is increased. The three parameters of the model are the barrier *U*_0_, the attempt rate τ_0_^–1^, and the prefactor time τ_c_.

## 3 Results and Discussion

We measure the friction of the C_60_ films using a quartz crystal microbalance. Details of this technique are described in Materials and Methods. Unlike previous studies,^30–32^ where the fullerene layers were initially thermally deposited on the QCM metal electrodes and then transferred to the sample cell for measurements, in our study, C_60_ is deposited in situ on one QCM electrode. The deposition of a film on the quartz electrode causes the resonance frequency *f*_0_ to decrease proportionally to the mass of the film, as illustrated in the scan of Figure 4a taken at high temperature, *T* = 355 K. Film coverage is deduced from the frequency shift assuming for the monolayer an areal density σ = 1.15 molecules/nm^2^, corresponding to a nearest-neighbor distance in closed packed layers of 1.0 nm, the same as the nearest-neighbor distance of the (111) face of the bulk fcc C_60_.^43,44^ This is equivalent to a frequency shift of 7.7 Hz for the adsorption of a complete monolayer. Furthermore, the first layer must essentially be completed before the molecules add to the second layer.^43^

**Figure 4:**
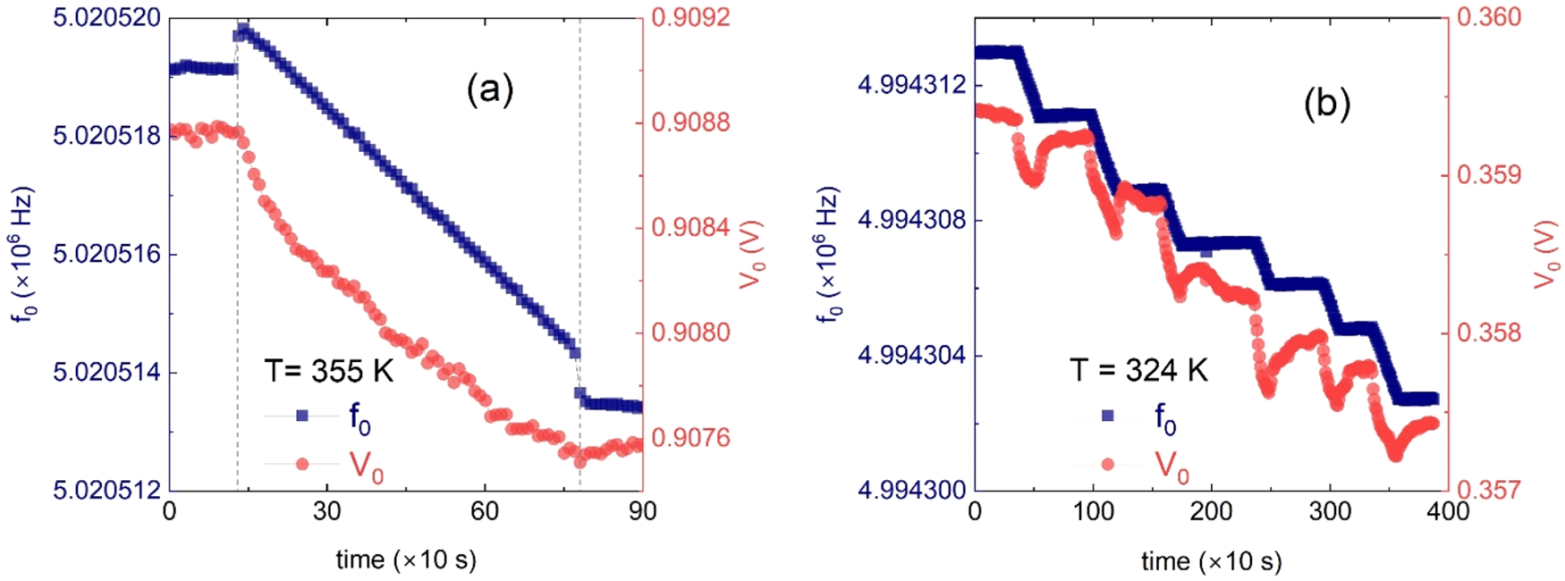
Dosing scans representing the resonance frequency (gray squares, left axes) and the amplitude (red circles, right axes) as C_60_ is deposited on Au(111). (a) The two jumps in resonance frequency observed at *T* = 355 K occur when the effusion cell shutter is open and closed, respectively. The overall frequency shift corresponds to a C_60_ coverage of about 0.75 monolayers. (b) The staircase curves at *T* = 324 K are due to repeated openings and closings of the shutter up to approximately 1.5 C_60_ monolayers. The corresponding values are taken by waiting for equilibrium to be reached after closing the effusion cell shutter at each step.

Figure 4a shows that it takes at least 20 min to complete the deposition of a nominal layer of C_60_. The scan of Figure 4a shows a sudden increase and decrease in frequency in correspondence to the opening and closing of the effusion cell shutter, respectively. This rise is due to the small temperature increase (approximately 0.1 K) caused by the exposure of the crystal to the hot source. We point out that exposure to the radiating heat from the evaporator can also cause a sudden change in the resonance amplitude *V*_0_, as shown in Figure 4b, at the moment when the dosing of C_60_ takes place. The actual response depends on the temperature dependence of the quartz resonance parameters. The scans reported in Figure 4 show two examples of the rich phenomenology we observed: a sudden increase in *f*_0_ at *T* = 355 K, see Figure 4a, and a sudden decrease in *V*_0_ at *T* = 324 K, see Figure 1b, after opening the shutter. However, the temperature response changes with the mounting of the quartz crystal in the custom case. We verified this by measuring the temperature response of the same quartz mounted in two different ways by varying the pressure exerted by the case screws.^38^ Consequently, to accurately determine *f*_0_ and *V*_0_, the slip data were taken in successive steps under thermal equilibrium conditions by depositing small fractions of the monolayer and waiting for the QCM to equilibrate. The resulting frequency scan exhibits the characteristic staircase shape exemplified by the scan taken at *T* = 323 K, shown in Figure 4b.

The scans of Figure 4 show that, concurring to the C_60_ deposition signaled by a decrease in the resonance frequency, there is a decrease in the resonance amplitude *V*_0_, which is due to any dissipation that occurs at the solid film interface.^45^ From the measured changes in the resonance frequency δ*f* and the amplitude δ*V* of the QCM, it is possible to determine^45^ the slip time $\tau_{s}=\frac{1}{4\pi }\frac{\delta V/V_{0}}{\delta f}$ . It represents the time constant of the exponential decay of the film velocity when the oscillating substrate is brought to a sudden stop. If τ_s_ = 0, the film is rigidly locked to the substrate, viceversa, if τ_s_ = ∞, the film is dynamically decoupled from the substrate as in the case of a superfluid film; for rare gases deposited at cryogenic temperatures on metallic surfaces, τ_s_ is found to be on the order of a few nanoseconds.^16,17^

Figure 5 summarizes the main results of the slip time τ_s_ of fullerene adsorbates deposited on a number of gold electrodes and at various temperatures. We point out that, for the sake of clarity, Figure 5 reports data from approximately half of the scans we acquired in the temperature interval of 273–363 K.

**Figure 5:**
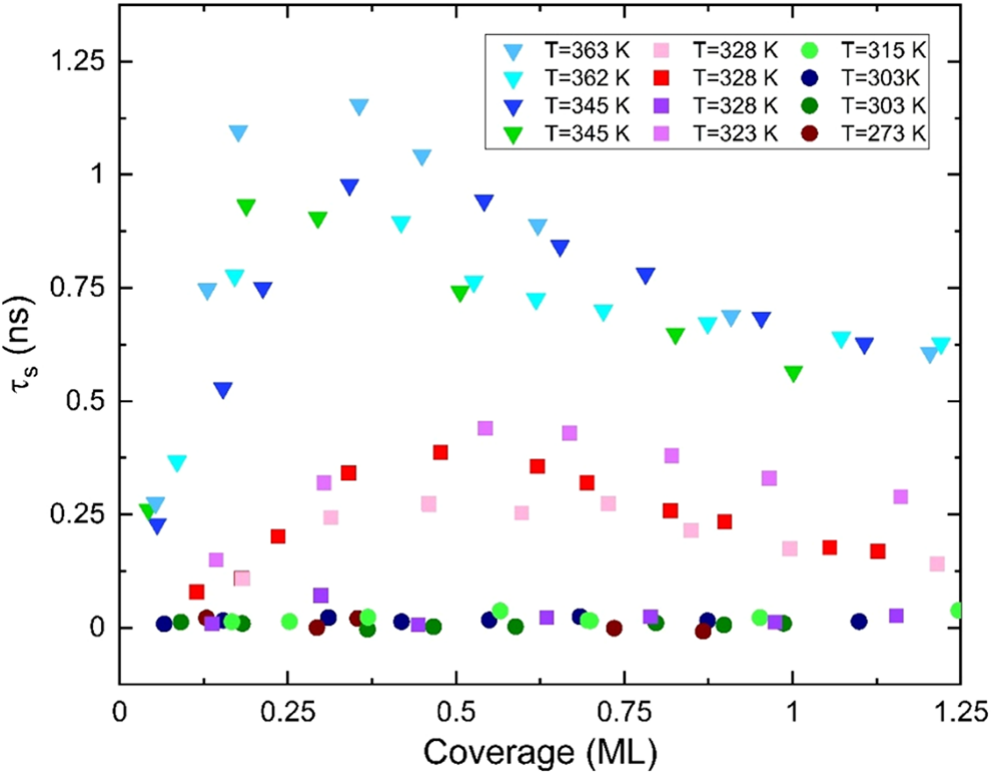
Slip time of C_60_ on Au(111) as a function of film coverage. The scans are taken at various temperatures, with all data taken at room temperature and below showing no slippage. Each scan refers to a gold layer deposited on a different quartz crystal.

Importantly, all data taken at room temperature and below shows no sliding, i.e., τ_s_ = 0. This observed pinning below a temperature threshold resembles qualitatively that observed for N_2_ monolayers on Pb surfaces at temperatures below 15 K.^46^ Instead, at higher temperatures (*T* ≳ 340 K), all the data exhibit slippage of C_60_ films, with characteristic slip times close to 1 ns, comparable to those reported for Xe on Au at cryogenic temperatures.^16^ At intermediate temperatures (*T ∼* 320 K), the data indicate either no or low slippage. The scatter observed in the collected experimental data at similar temperatures and coverage can be reasonably ascribed to variations in the quality of the surfaces of the gold electrodes used.

Focusing on the probed higher-temperature range in Figure 5, with clear finite values of τ_s_ signaling slippage, the QCM data show thermal depinning with an initial rapid increase in slip time as a function of the adsorbate coverage θ, reaching typical QCM peak values (τ_*s*_ ∼ 1 ns) at around θ = 0.4 ML.^17^ The large and rapid rise of τ_s_ with coverage may signal the presence of a partially finite-size incommensurate interface, whose pinning, mainly due to the edges of the C_60_ islands, scale sublinearly with the adsorbate mass;^20^ the imposed QCM driving force, instead, increases linearly with the adsorbate coverage. Beyond θ ∼ 0.4 ML, individual C_60_ islands, whose average size is likely to increase with adsorbate coverage, eventually become comparable in size with flat Au(111) terraces. As a result, larger islands are more likely to interact significantly with and be pinned by substrate imperfections, such as the atomic step edges visible in Figure 2, determining a gradually decreasing trend of the measured slip time as a function of θ.

Figure 6 explicitly highlights the temperature dependence of the slip times measured by QCM at a nominal C_60_ film coverage θ = 0.25 ML and its good agreement with the proposed thermally activated diffusive model described in Materials and Methods. Ab-initio calculations of single-molecule C_60_/Au­(111) energetics,^35,39,40^ although disagreeing on important details such as the precise adsorption site and highlighting complications related to the surface reconstruction of gold and the dynamics of the top Au layer, determine lateral corrugation barriers against translation roughly in the 100 meV region. However, at nonnegligible surface coverage, C_60_ molecules will mostly cluster together, thus forming aggregate islands with presumably larger barriers against translation.^47,48^ Therefore, we take the energy barrier itself as a fitting parameter of the model. A best fit of the experimental slip times of Figure 3 yields the three model parameters: the barrier *U*_0_ = 333 meV, the attempt rate τ_0_^–1^ = 5.48 × 10^11^ Hz, and the prefactor time τ_c_ = 1.11 ns. The resulting model curve perfectly fits the data. As pointed out in the previous section, adopting a different s-shaped function, for example $s\left(x\right)=\frac{x^{8}}{1+x^{8}}$ leads to an analogous fit quality, and very close best-fit parameters, with a barrier *U*_0_ = 332 meV.

**Figure 6:**
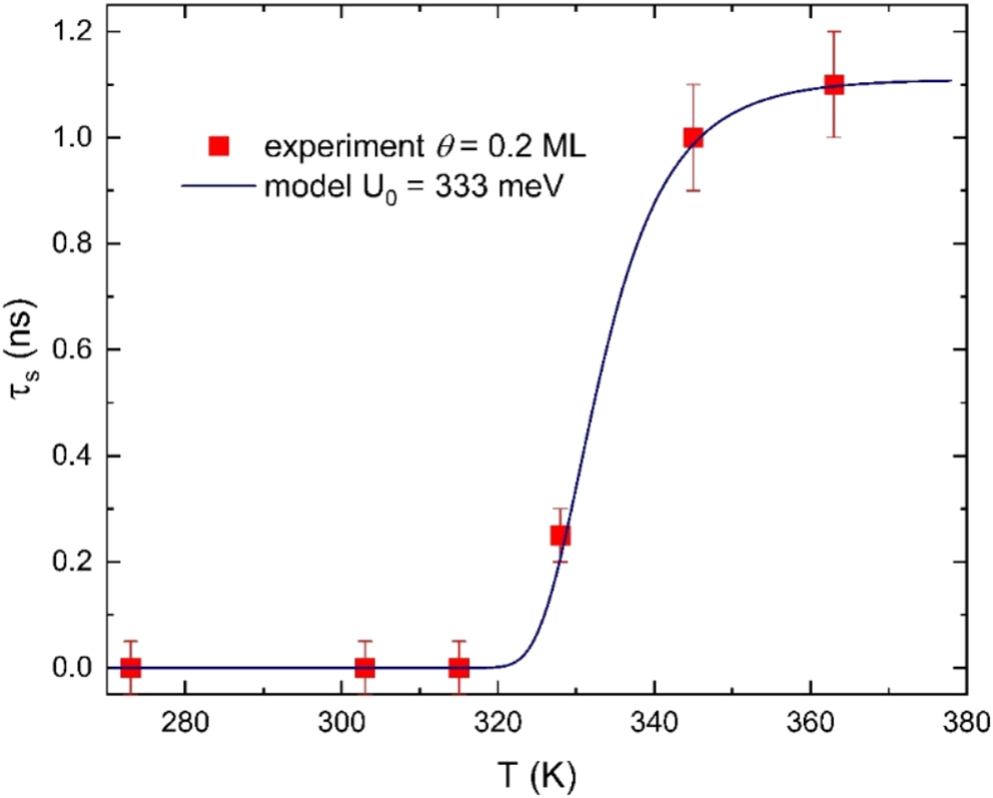
Temperature dependence of the slip time corresponding to a C_60_ film coverage θ = 0.25 ML. The continuous blue line is the best fit to the experimental data calculated according to the thermally activated diffusive model described in the text. In particular, the energy barrier against lateral motion is found to be *U*_0_ = 333 meV.

## 4 Conclusions

With a quartz crystal microbalance, we have measured the depinning of C_60_ monolayers on high-quality gold electrodes as the temperature is raised above room temperature. We have observed a sharp increase in slip time with temperature. At the highest temperatures explored in our study, the measured slip times are on the order of 1 ns, comparable to those commonly observed for light atomic gases. This result may seem surprising in view of the strong attraction of heavy C_60_ molecules to the gold surface (adhesion energy estimated in the 1–2 eV region^39,40^ ), suggesting a nearly covalent interaction between C_60_ and gold. However, the slip time is mainly determined by the low-energy corrugation against the lateral motion of C_60_, an indication of the slipperiness of the carbon–gold contact, and whose amplitude is further reduced effectively by thermal effects and interface incommensurability. Vacancy diffusion in the gold top surface layer, which was suggested in ref 35, may perhaps play a role in the abrupt unpinning of the C_60_ layer observed. Dedicated experiments (e.g., STM at elevated temperatures) are required to validate this hypothesis. To the best of our knowledge, this is the first observation of the sliding of heavy molecules obtained with the quartz crystal microbalance, which so far has been applied to the investigation of the nanofriction of light atoms and molecules at cryogenic temperatures. Our results suggest that QCM can be an effective probe for addressing molecular friction, providing information that is complementary to friction force microscopy.
